# Gastrointestinal tract metastasis presenting as intussusception in invasive lobular carcinoma of the breast: A case report

**DOI:** 10.1016/j.ijscr.2019.10.003

**Published:** 2019-10-07

**Authors:** Joanne Aisha Mosiun, Muhammad Syafiq bin Idris, Li Ying Teoh, Mei Sze Teh, Patricia Ann Chandran, Mee Hoong See

**Affiliations:** a Department of General Surgery, University Malaya Medical Centre, Lembah Pantai, 59100, Kuala Lumpur, Malaysia; b Department of Pathology, University Malaya Medical Centre, Lembah Pantai, 59100, Kuala Lumpur, Malaysia

**Keywords:** Breast cancer, Invasive lobular carcinoma, Gastrointestinal metastasis, Intussusception, Case report

## Abstract

**Introduction:**

Breast cancer metastasis to the gastrointestinal (GI) tract is rare and occurs more frequently in invasive lobular carcinoma. Patients may be asymptomatic or present with variable vague symptoms that may be mistakenly attributed to side effects of chemotherapy or other benign GI diseases. Treatment follows the principles of systemic disease and includes hormonal therapy, chemotherapy and signal transduction inhibitors, with surgical intervention indicated for complications such as obstruction, perforation and hemorrhage.

**Presentation of case:**

We present the case of a female patient with a history of invasive lobular breast carcinoma who had undergone mastectomy and axillary dissection, followed by chemoradiotherapy. Over the next nine years, she developed ovarian and bone metastases for which appropriate treatment was provided. A right iliac fossa mass was discovered during routine clinic review, though she remained asymptomatic. Computed tomography scan showed ileocecal intussusception. Histopathological examination of the right hemicolectomy specimen following emergency surgery confirmed metastatic invasive lobular carcinoma to the GI tract.

**Discussion:**

GI tract metastasis may present 30 years after the primary breast cancer. Up to 20% of patients may be asymptomatic as shown by Montagna et al. When present, symptoms are commonly non-specific and vague. Histological diagnosis is challenging. GI metastasis typically appears as intramural infiltration of the bowel wall by small cells arranged in cords.

**Conclusion:**

It is important to maintain a suspicion for GI tract metastasis in breast cancer patients who present with abdominal mass or GI symptoms, as this aids in prompt institution of accurate and appropriate management.

## Highlights

• Gastrointestinal (GI) tract metastasis in breast cancer is rare and occurs more commonly in invasive lobular carcinoma.
• The interval between the index breast cancer and GI tract spread may be as long as 30 years.
• 20% of patients with GI tract metastasis are asymptomatic, and detection may rely on physical examination and imaging.
• Management follows the principles of treatment in systemic disease in breast cancer, with consideration for surgery in obstruction, perforation or bleeding.

## 1 Introduction

Gastrointestinal (GI) tract metastasis in breast cancer is rare, with autopsy studies reporting an incidence of 6–18% [1]. Compared to invasive ductal carcinoma, the lobular subtype has a predilection for distant spread to unusual sites such as the GI tract, peritoneum and adnexa [2]. Any part of the GI tract may be affected [3]. The most commonly involved sites are the stomach (6–18%), followed by the colon or rectum (8–12%). Metastasis to the small bowel seldom occurs [4].

Patients with GI tract metastasis may remain asymptomatic, present with non-specific symptoms or manifest with intestinal obstruction, perforation or bleeding. GI tract metastasis may occasionally be the first indicator of an undiagnosed breast cancer [5]. Schwarz et al. has reported a median interval of 6 years (range 0.25–12.5 years) between the diagnosis of breast cancer and the development of GI tract secondary tumors [6]. However, a cancer-free interval of as long as 30 years has been reported by Benfiguig et al. [7].

The subtle presentation of GI tract metastasis may mimic other benign GI conditions. Thus, a high index of suspicion should be maintained to avoid delayed diagnosis and treatment. Management follows the principles of systemic disease and includes hormonal therapy, chemotherapy and signal transduction inhibitors. Palliative surgery is indicated in patients who present with complications of GI tract metastasis.

This work has been reported in line with the SCARE criteria [8].

## 2 Presentation of case

A 51-year-old female underwent right mastectomy and axillary dissection in 2008 for Stage 3 (T2N2M0) invasive lobular carcinoma of the right breast which was negative for estrogen (ER) and progesterone receptors (PR), and equivocal for human epidermal growth factor receptor 2 (HER2). Histopathological examination (HPE) of the mastectomy specimen showed clear margins with no lymphovascular invasion. She completed three cycles of 5-fluorouracil, epirubicin and cyclophosphamide (FEC), and three cycles of taxotere. This was followed by 15 fractions of radiotherapy to the right chest wall and supraclavicular fossa.

Two years later, the patient developed metastatic lobular carcinoma to the ovaries. She underwent total abdominal hysterectomy and bilateral salpingo-oophorectomy. The metastatic tumor was positive for ER and PR, and equivocal for HER2. She was subjected to six cycles of chemotherapy comprising gemcitabine and cisplatin, after which tamoxifen was initiated. Seven years later, a left breast lump with palpable axillary and supraclavicular lymph nodes were found. Biopsy of the lump revealed invasive lobular carcinoma, ER and PR positive, and equivocal for HER2. Computed tomography (CT) detected bone metastasis. Daily oral letrozole led to resolution of both breast lump and lymph nodes. She was started on monthly intravenous zoledronic acid injections.

**Fig. 1:**
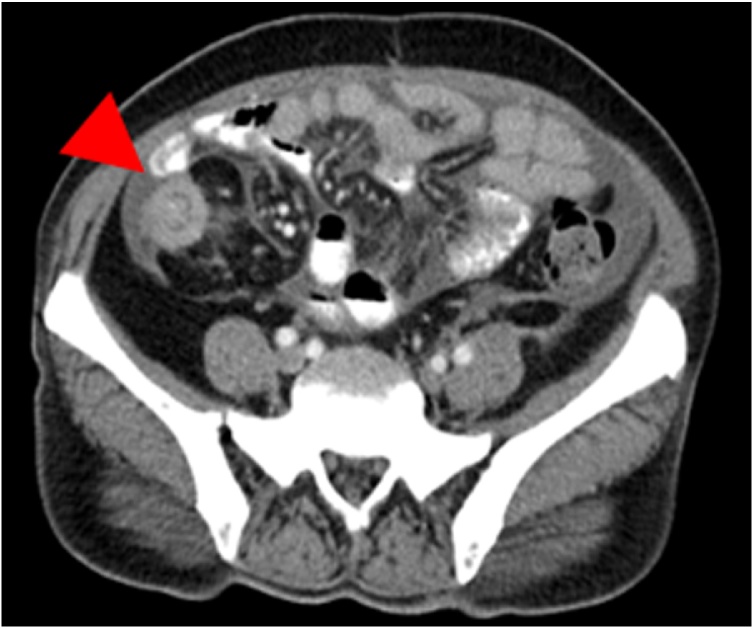
Axial section of the abdominal CT scan demonstrating the target sign of intussusception, marked by a red arrowhead.

**Fig. 2:**
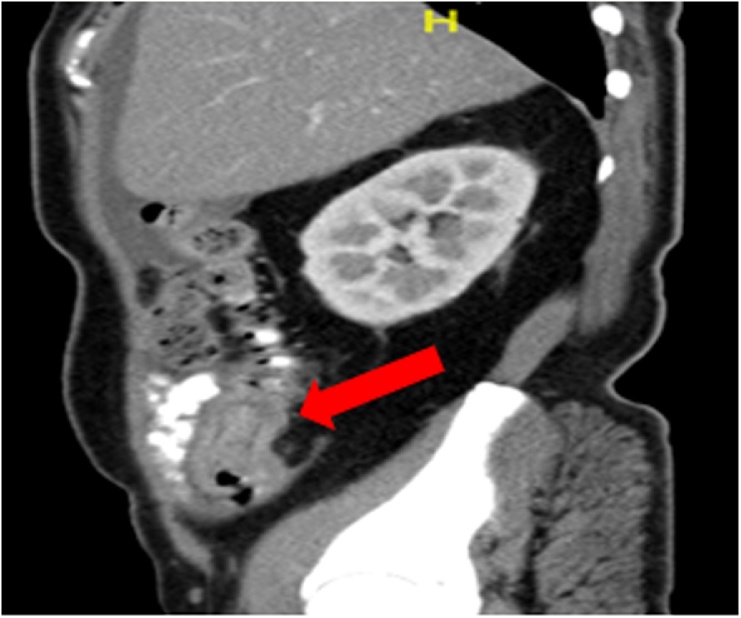
Sagittal section of the abdominal CT scan showing an entrapped bowel segment within an edematous outer bowel loop, marked by a red arrow. The proximal small bowel was not dilated.

Seven months later, the patient was found to have a right iliac fossa mass on abdominal examination. She denied having abdominal pain, vomiting, altered bowel habit or passage of bloody stools. There were no signs or symptoms or intestinal obstruction. A contrasted abdominal CT showed ileocecal intussusception (Fig. 1, Fig. 2).

**Fig. 3:**
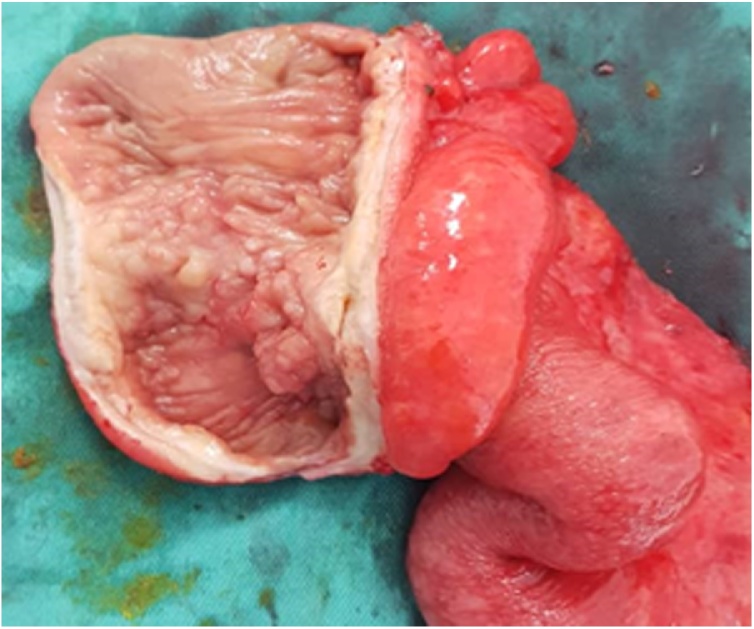
Sectioning of the right hemicolectomy specimen revealed telescoping of the distal ileum into the cecum through the ileocecal valve. The terminal ileum wall was thickened and fibrotic with whitish solid nodules. Multiple small polyps were seen in the cecum and part of the ascending colon mucosa.

The patient underwent emergency exploratory laparotomy. Intraoperatively, one litre of straw-colored ascitic fluid was drained from the peritoneal cavity. There were multiple peritoneal nodules as well as enlarged intraabdominal lymph nodes. Right hemicolectomy with the creation of double barrel stoma was performed (Fig. 3).

**Fig. 4:**
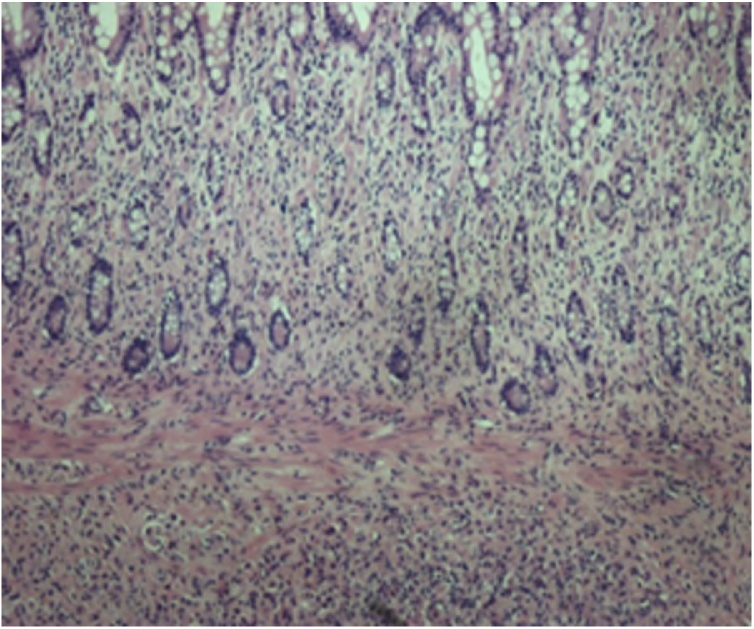
Microscopic examination of the resected specimen at low power magnification (40×) showed diffuse infiltration of the submucosa and lamina propria right up to the mucosal layer in single file pattern.

**Fig. 5:**
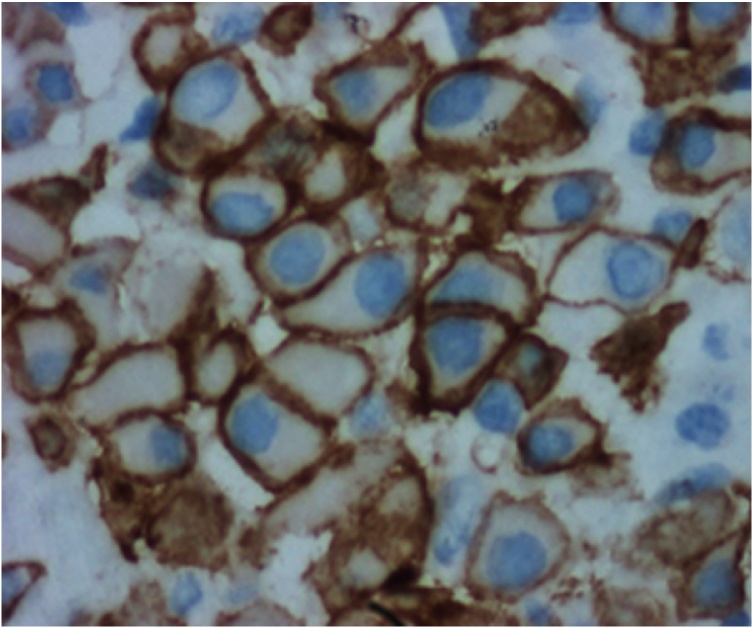
The tumor cells were round to oval in shape with nuclear pleomorphism and vesicular nuclei. There was HER2 overexpression in the cytoplasmic membrane of malignant cells.

Microscopically, metastatic invasive lobular carcinoma with tumor cell infiltration into the full thickness of the terminal ileum, cecum and appendix was seen. Both proximal and distal surgical margins were involved and tumor deposits were seen in the sampled lymph nodes. There was weak ER positivity and focal staining for PR. HER2 was focally overexpressed. The tumor cells stained negative for E-cadherin (Fig. 4, Fig. 5).

Post-operative recovery was uneventful. The patient was continued on oral letrozole and intravenous zoledronic acid injections. She could not afford CDK4/6 inhibitor treatment due to financial constraints. Palliative paclitaxel was started three months later due to disease progression with malignant ascites.

## 3 Discussion

Invasive lobular carcinoma is a distinct subtype of invasive breast cancer that accounts for 5–15% of breast cancer cases [9]. Despite the higher prevalence of invasive ductal carcinoma, the lobular subset has been found to metastasize to the GI tract more frequently. In a study conducted by Borst et al. involving a series of 2605 patients, it was found that there was a statistical difference in the incidence of GI tract metastasis between invasive ductal and lobular carcinoma. The incidence of GI tract spread in invasive lobular carcinoma was 4.5% compared to 0.8% in invasive ductal carcinoma [10]. In patients with mixed ductal and lobular carcinoma, the likely cause of the metastatic disease is commonly the lobular component [1].

The reason for a higher rate of GI tract metastasis in invasive lobular carcinoma compared to ductal carcinoma has not been fully elucidated. However, there are postulations that it involves the loss of E-cadherins and the microenvironment of the GI tract. E-cadherins are cell-to-cell adhesion molecules that play a role in maintaining differentiation and preventing invasion. Invasive lobular carcinoma stains negative for E-cadherin. Consequently, this subtype of breast cancer is more dedifferentiated and invasive [11]. The microenvironment of the GI tract is thought to be conducive to the growth of secondary tumor cells as it provides the required building blocks for tumor proliferation, as well as possessing a microanatomy that favors entrapment of tumor cells [12].

Our patient did not have any GI complaints and was only incidentally found to have an abdominal mass on examination. A case series of 40 patients by Montagna et al. showed that 80% of patients presented with GI symptoms, while the remaining 20% were asymptomatic [13]. This concurred with the findings of Switzer et al. in a case series of 21 patients which showed that 20% of patients were asymptomatic. Symptomatic patients presented with nausea (20%), abdominal pain (15%), small bowel obstruction (10%), dysphagia (5%), and GI bleed (5%) [14]. A review of 32 patients who sought treatment for GI tract breast cancer metastasis at the Mayo Clinic in Rochester from 2000 to 2013 showed that common presentations were nausea or vomiting (56%) and changes in stool (28%). Other complaints included weight loss, anemia, ascites, early satiety and dysphagia. Four patients (12%) were asymptomatic with metastases found incidentally on routine scanning [15].

Histological diagnosis of GI tract metastasis in breast cancer can be challenging. GI tract metastasis of lobular carcinoma typically shows an intramural infiltration involving the serosal, muscular and submucosal layers with small cells invading the tissue in cords [16]. The tumor cells frequently have a signet-ring appearance [17]. A feature suggestive of metastatic spread is the lack of dysplasia or atypia in the adjacent colonic epithelium and the visualization of tumor cells infiltrating the nearby native preexisting glands. Immunohistochemical (IHC) markers are helpful in the diagnosis of metastatic lobular breast carcinomas. Cytokeratin 7 (CK 7), ER, PR and gross cystic disease fluid protein-15 (GCDFP-15) usually stain positive in secondary deposits of lobular carcinoma and negative in primary GI tumors. Cytokeratin (CK 20) and carcinoembryonic antigen (CEA) are usually positive in primary GI tumors and negative in metastatic lobular carcinoma to the GI tract [18]. The IHC staining of this patient’s hemicolectomy specimen showed that there was a weak positivity for ER and focal staining of PR. There was also overexpression of HER2. This pattern of IHC staining was favorable for the diagnosis of metastatic lobular breast carcinoma.

The treatment for metastatic breast cancer with GI tract involvement is systemic treatment with chemotherapy, endocrine therapy or signal transduction inhibitors. Palliative surgery is necessary in patients with complications such as intestinal obstruction, perforation or hemorrhage. Remissions are observed in 32–53% of patients [19]. Switzer et al. found that the five-year survival from diagnosis of GI tract metastasis was 29% [14].

## 4 Conclusion

In conclusion, patients with a history of breast cancer who present with GI symptoms should be investigated for GI tract metastasis, regardless of how long ago the primary breast cancer diagnosis was made. Early investigation and establishment of the diagnosis are vital in ensuring prompt and adequate treatment.

## Sources of funding

None to declare.

## Ethical approval

This case report is exempt from ethical approval by our institution.

## Consent

Written informed consent was obtained from the patient for publication of this case report and accompanying images. A copy of the written consent is available for review by the Editor-in-Chief of this journal on request.

## Author contributions

Joanne Aisha Mosiun: Conceptualization, Acquisition of data, Writing - original draft preparation.

Muhammad Syafiq bin Idris: Surgical therapy for this patient, Visualization, Writing - Reviewing and Editing, Supervision.

Teoh Li Ying: Writing - Reviewing and Editing, Supervision.

Teh Mei Sze: Writing - Reviewing and Editing, Supervision.

Patricia Ann Chandran: Visualization, Writing - Reviewing and Editing.

See Mee Hoong: Writing - Reviewing and Editing, Supervision, Validation.

## Registration of research studies

None.

## Guarantor

See Mee Hoong.

## Provenance and peer review

Not commissioned, externally peer-reviewed.

## Declaration of Competing Interest

No potential conflicts of interest.
